# Association between Diabetes and Levels of Micronutrients in Qatar—A Case–Control Study

**DOI:** 10.3390/biomedicines11113045

**Published:** 2023-11-14

**Authors:** Nada Soliman, Ruba Almishal, Basant Elsayed, Ayaaz Ahmed, Sara Al-Amri, Aisha Al-Kuwari, Shaikha Al-Muhannadi, Muhammed Nadeer, Tawanda Chivese

**Affiliations:** College of Medicine, QU Health, Qatar University, Doha P.O. Box 2713, Qatar; ns1706472@qu.edu.qa (N.S.); ra1605902@qu.edu.qa (R.A.); be1802020@qu.edu.qa (B.E.); aa1703722@qu.edu.qa (A.A.); sa1602153@qu.edu.qa (S.A.-A.); aa1602749@qu.edu.qa (A.A.-K.); sa1509132@qu.edu.qa (S.A.-M.); mn1703939@qu.edu.qa (M.N.)

**Keywords:** diabetes mellitus, diabetes control, microelements, macroelements, vitamins, magnesium, Qatar Biobank (QBB)

## Abstract

Objective: The objectives of this study were to investigate associations between micronutrient levels and diabetes and to explore the association in individuals with controlled and uncontrolled diabetes. Methods: A case–control study, matched on age and gender, was performed on participants with (cases) and without diabetes (controls), who were Qatari or long-term residents (≥15 years of residence). Participants with diabetes were divided into those with controlled and uncontrolled diabetes using an HbA1c cutoff of 7%. Levels of micronutrients were measured from serum and categorized into normal and abnormal levels. Results: A total of 1118 participants (374 cases and 744 controls) were included with a mean age of 41.7 years (SD 9.9), of whom 53.9% were female. Of those with diabetes, 229 had controlled diabetes and 145 had uncontrolled diabetes. Compared to those without diabetes, participants with diabetes had significantly lower mean magnesium (0.80 mmol/L (SD 0.07) vs. 0.84 mmol/L (SD 0.06), respectively, *p* < 0.001). Lower magnesium and iron were observed in participants with uncontrolled compared to participants with controlled diabetes. After multivariable logistic regression, diabetes was associated with hypomagnesemia (OR 3.2, 95% CI 3.4–213.9) and low iron (OR 1.49, 95% CI 1.03–2.15). Uncontrolled diabetes showed stronger odds of association with hypomagnesemia (OR 5.57, 95% CI 3.65–8.52). Conclusion: In an affluent setting in the MENA region, diabetes was associated with low magnesium and low iron, and this association was stronger in individuals with uncontrolled diabetes.

## 1. Introduction

Diabetes mellitus is one of the most common diseases in the world and has a negative impact on the health of populations and individuals, evidenced by its significant contribution to mortality and morbidity [1]. The majority of people with diabetes have type 2 diabetes, followed by gestational diabetes and type 1 diabetes, and a smaller proportion has other forms of diabetes [1,2,3,4]. Notably, type 2 diabetes is preventable, and emerging evidence suggests that the disease is also reversible in some subgroups of affected people [5]. The worldwide prevalence of diabetes mellitus is increasing and expected to rise from 537 million in 2021 to 784 million by 2045 [1]. The Middle East and North Africa (MENA) region has one of the highest age-adjusted comparative prevalence rates of diabetes in people aged 20–79 years, at 16% [1]. Qatar, a country within the MENA region, is one of the countries that is highly affected by diabetes. With a prevalence of diabetes of around 16.4%, the state of Qatar has the highest expenditure for diabetes per person, compared to other nations, at approximately USD2017.00 per person per annum [1]. Most of the healthcare costs associated with diabetes are related to treatment to maintain blood glucose levels within safe physiological units, although medical and surgical weight reduction therapies are increasingly being accepted as diabetes therapy, especially for individuals with type 2 diabetes [1,6]. Uncontrolled diabetes is associated with a higher risk of complications and the need for frequent hospitalizations, both of which are associated with a higher cost of diabetes care [1]. Therefore, there is a compelling need to explore factors that may affect both the development of diabetes and achieve better control of diabetes in Qatar and in the wider MENA region.

Alterations in micronutrient intake and serum levels are factors that have been implicated in both the development and control of diabetes [7]. Micronutrients are nutrients that the body requires in miniscule amounts and can be divided into the following categories: macroelements, microelements (or trace elements), and vitamins [8]. These elements are involved in various processes related to glucose metabolism and thus have been targeted as possible preventive or management options for diabetes.

Macroelements, such as calcium (Ca), magnesium (Mg), and iron (Fe), are elements required by adults in amounts exceeding 100 mg/day, while microelements like zinc (Zn) and copper (Cu) are needed in amounts between 1 and 100 mg/day [9]. Mg is involved in glucose metabolism [10], too much Fe can cause oxidative damage to β-cells, and Zn is crucial in insulin production [7]. Vitamins also contribute to many different biological processes and have attracted some interest either as preventive or therapeutic agents for diabetes [11]. Vitamin B9 (folate) and vitamin B12 aid in the conversion of homocysteine to methionine, thus reducing oxidative damage, which is common in diabetes and frequently associated with peripheral neuropathy [12].

Despite the possible links between micronutrients and glucose metabolism, findings from both observational and experimental studies about their role in the development and control of diabetes have largely been heterogeneous. Several meta-analyses of randomized controlled trials (RCTs) have produced conflicting results about the benefits of supplementation with micronutrients in individuals with diabetes, with some showing benefits in reducing diabetes risk and achieving better control of diabetes, while others showed no benefit [13,14,15]. Therefore, there is still a further need to investigate the relationship between micronutrient levels and diabetes, as well as how these micronutrients play a part in the control of diabetes. In the MENA region, only a handful of studies have investigated the relationship between diabetes and the levels of micronutrients. Given the high prevalence of diabetes in the MENA region and well-established deficiencies of some micronutrients such as vitamin D [16], there is a need to study the impact of micronutrient deficiencies and both the risk of diabetes and control of diabetes. The prevalence of deficiencies in many micronutrients is not well studied in Qatar, and this is also true of the relationship between the prevalence of these nutrients and both diabetes risk and diabetes control. This study aimed to compare the levels of micronutrients between individuals with and without diabetes. We also investigated the association between these micronutrients in stratified groups of individuals with uncontrolled diabetes and those with controlled diabetes.

## 2. Methods

### 2.1. Design, Population, and Setting

A case–control study, using data collected by Qatar Biobank (QBB), was conducted on Qatari citizens and long-term residents. Long-term residents were defined as individuals who have lived in the state of Qatar for at least 15 years. The QBB is a population-based research initiative that provides a depository of biological samples and information on the health and lifestyle of Qatari citizens and long-term residents [17,18]. The QBB aims to prospectively recruit 60,000 participants and has so far collected data from more than 30,000 individuals [17,18]. The design of the QBB follows an agnostic-hypothesis (i.e., not hypothesis-driven) cohort, whereby participants give data and samples on wide-ranging exposures and outcomes, similar to the United Kingdom Biobank [17,18]. These data include socio-demographic information; medical and surgical history; current medical conditions; laboratory measurements; blood, urine, and saliva samples; genetics; radiological data from X-rays; magnetic resonance imaging (MRI); and diet and physical activity information, among others [17,18]. Some of the measurements are carried out on randomly selected individuals to reduce costs. Participants from the QBB, above 18 years of age with data on micronutrients were eligible for inclusion in the present study. During this study, participants with diabetes were randomly selected from the QBB database and matched, using age, gender and nationality, to those without diabetes. Participants without data on micronutrients, those aged below 18 years, and pregnant women were excluded.

### 2.2. Data Collection

When they participated at the QBB, each participant completed a questionnaire, had anthropometry measured, and had blood drawn for the measurement of serum micronutrients. For the current study, we requested the QBB to provide data for each participant on demographics, data on self-reported doctor-diagnosed diabetes, duration of diabetes, nutritional supplement usage, income, smoking status, blood pressure, anthropometry, HbA1c level, serum levels of lipids, random glucose, and micronutrients. The micronutrients that the QBB measured were copper (Cu), zinc (Zn), magnesium (Mg), iron (Fe), folate, and vitamin B12. Notably, some of the micronutrient measurements were introduced in later years after the establishment of the QBB, and, at the time of this study, only a few participants had data on such micronutrients. This applied to copper and zinc, in particular.

### 2.3. Diabetes Measurements

Both diabetes diagnosis and diabetes control were defined using the American Diabetes Association (ADA) criteria of 2021 [19]. Participants were classified into the diabetes group if they self-reported being diagnosed by a doctor or if they had an HbA1c ≥6.5% or a random blood glucose level ≥11.1 mmol/L, according to the ADA criteria [19]. Controlled diabetes was defined based on the ADA guidelines as HbA1c ≤ 7% [19].

### 2.4. Micro- and Macroelements and Vitamin Measurements

For each participant, micronutrients were assessed from their serum measurements. Levels of micronutrients were classified as ‘low’ according to the normal laboratory ranges for adults on UpToDate [20], using the following cut-offs: Mg (≤0.66 mmol/L), Fe (≤9 μmol/L), Zn (≤11.5 μmol/L), Cu (≤15.7 μmol/L), folate (≤4.1 nmol/L), and vitamin B12 (≤147.5 pmol/L).

### 2.5. Ethics and Informed Consent

This study used data that were already collected by the QBB, and therefore, informed consent was not required (since it was already given). At the QBB, participants gave written informed consent before data were collected. The study received ethical approval and a waiver of informed consent from the Qatar University’s Institutional Review Board (QU-IRB) (reference number: QU-IRB1228-E/20) and the Qatar Biobank’s IRB (reference number: QF-QBB-RES-ACC-0186). The study was conducted in accordance with the ethical principles for medical research that involves humans of the Declaration of Helsinki [21]. All the investigators undertook training in and adhered to good clinical practice during the research.

### 2.6. Data Analysis

Histograms and the Shapiro–Wilks test were used to check for the normality of data distribution for numerical variables. Descriptive data were presented as means and standard deviations (SDs) if normally distributed, or as medians and interquartile ranges (IQRs) if not normally distributed. Categorical variables were summarized using frequencies and percentages. Boxplots were used to graphically present comparisons of the levels of the micronutrients between participants without diabetes, those with controlled diabetes, and participants with uncontrolled diabetes. The micronutrient levels were compared between participants with and without diabetes using the *t*-test for independent groups if normally distributed or the Wilcoxon rank-sum test if not normally distributed. Comparisons of micronutrient levels between the three groups (controlled, uncontrolled, and no diabetes) were tested using one-way ANOVA if the data were normally distributed, or the Dunn test if the data were not normally distributed with a post hoc test carried out using Bonferroni adjustment.

Multivariable logistic regression was carried out for the association between diabetes and the micronutrients, with separate models for each micronutrient and diabetes (yes/no) as a binary outcome. To investigate the association between micronutrients and uncontrolled diabetes, multivariable multinomial logistic regression was used, with “no diabetes” as the base outcome. In all models, we adjusted for age, gender, BMI, smoking, and income, as they are known to be associated with both micronutrients’ intake and diabetes, and directed acyclic graphs were then used to decide on the appropriate confounders [22,23,24]. For each multivariable regression model, we tested the model specification using the linktest. Exact *p*-values were reported and interpreted as evidence against the null hypothesis, and 95% confidence intervals (95% CI) were reported for odds ratios (ORs). All data analyses were performed using Stata 16.0 statistical software (College Station, TX, USA).

## 3. Results

### 3.1. Characteristics of Participants by Diabetes Status

A total of 1118 participants, of which 33.5% had diabetes, were included in the study. Participants with diabetes were slightly older than those without (mean age 44.4 years old (9.4) vs. 44.3 years old (9.9), *p* < 0.001). Participants with diabetes compared to those without had significantly higher mean BMIs (31.5 kg/m^2^ (SD 5.4) vs. 29.8 kg/m^2^ (SD 5.6), respectively, *p* < 0.001) (Table 1). Participants with diabetes, compared to those without diabetes, were also more likely to have tertiary education (Table 1). The median HaA1C, mean BMI, and mean waist-to-hip ratio were also significantly higher in participants with diabetes, compared to those without diabetes, as expected (Table 1). In relation to the lipid profile, in participants with diabetes, the mean HDL-cholesterol was lower, and LDL-cholesterol was significantly higher compared to that in individuals without diabetes, while total cholesterol levels were similar (Table 1). The proportions of individuals with dyslipidemia, hypertension, and a family history of diabetes were also consistently higher in the individuals with diabetes compared to those without diabetes (Table 1).

### 3.2. Comparison of Characteristics by Controlled Diabetes Status

As expected, the median HbA1C was higher in the participants with uncontrolled diabetes, compared to those with controlled diabetes (7.8% (IQR 7.4–8.9) vs. 6.0% (IQR 5.5–6.4), respectively, *p* < 0.001) (Table 1). The median duration of diabetes for participants with uncontrolled diabetes was longer compared to the participants with controlled diabetes (7 years (IQR 4–13) vs. 4 years (IQR 2–9), respectively, *p* < 0.001). A greater percentage of participants with controlled diabetes reported taking multivitamins/mineral supplements compared to those with uncontrolled diabetes (51.1% vs. 45.0%, respectively, *p* = 0.011).

### 3.3. Comparison of Micronutrient Levels between Participants with and without Diabetes

The median serum levels of folate were significantly higher in participants with diabetes compared to those without (Table 2). The median Mg serum levels were significantly lower in participants with diabetes, compared to those without diabetes (0.79 mmol/L (SD 0.07) vs. 0.84 mmol/L (SD 0.06), *p* < 0.001). For categorized micronutrients, compared to participants without diabetes, a significantly higher proportion of participants with diabetes had low magnesium (48.9% vs. 22.3%, *p* < 0.001) (Table 2).

**Figure 1:**
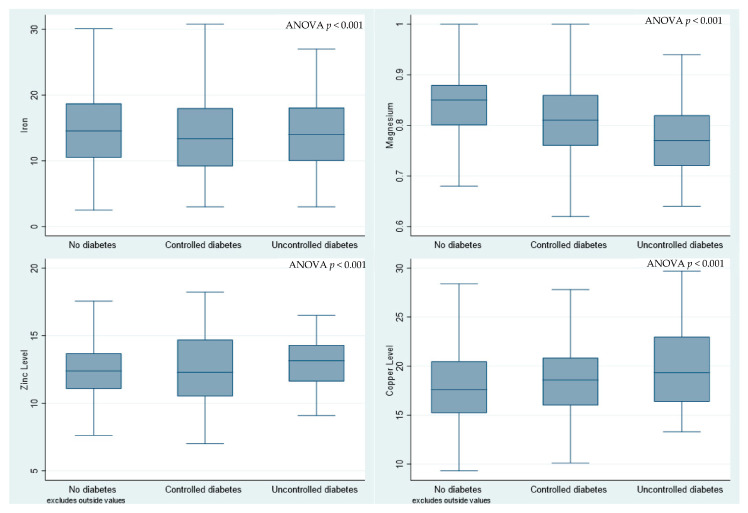
Boxplots comparing selected micronutrent levels across the three groups.

The boxplots in Figure 1 and Table 2 show a comparison of the levels of micronutrients between participants without diabetes, those with controlled diabetes, and participants with uncontrolled diabetes. The mean Mg level was significantly higher in participants with controlled diabetes compared to those with uncontrolled diabetes (0.81 mmol/L (SD 0.07) vs. 0.76 mmol/L (SD 0.07), respectively, *p* < 0.001)) (Table 2 and Figure 1). The median vitamin B12 levels were significantly higher in participants with uncontrolled diabetes compared to participants with either controlled or without diabetes (Table 2). There were no significant differences between the three groups in the remaining micronutrients (Table 2). The median serum levels of iron were significantly lower in participants with diabetes compared to those without (Table 2).

### 3.4. Association between Diabetes and Micronutrients—Multivariable Logistic Regression

After multivariable logistic regression, diabetes, compared to no diabetes, was significantly associated with 3-fold odds of having low magnesium (OR 3.32, 95% CI 2.47–4.47, *p* < 0.001) and a 49% increase in the odds of having low iron (OR 1.49, 95% CI 1.03–2.16, *p* = 0.034). Diabetes was also associated with a 49% increase in the odds of having low Zn, although with weak evidence against the null hypothesis (*p* = 0.334) (Table 3).

### 3.5. Association between Micronutrients and Controlled Diabetes—Multivariable Multinomial Logistic Regression

In further stratified analyses, uncontrolled diabetes was associated with 5.6-fold increased odds of having low magnesium (OR 5.57, 95% CI 3.65–8.57, *p* < 0.001) and 2-fold increased odds of having low iron (OR 1.75, 95% CI 1.01–3.03, *p* = 0.047). These associations were still observed in participants with controlled diabetes, but they were noticeably weaker (Table 3). Lastly, in participants with controlled diabetes, diabetes was associated with a two-and-a-half-fold increase in the odds of having low Zn, although there was weak evidence against the null hypothesis with the study’s sample size (Table 3).

## 4. Discussion

In this case–control study, we found lower serum levels for Mg and Fe in participants with diabetes, compared to those without diabetes. After adjusting for confounders, low magnesium and low iron were significantly associated with diabetes. These associations showed higher effect magnitudes in participants with uncontrolled diabetes compared to those with controlled diabetes.

Low magnesium was significantly associated with a 3-fold increase in diabetes risk and a 6-fold increase in the risk of uncontrolled diabetes. Consistent with our finding, one study showed Mg levels were lower in uncontrolled diabetes compared to controlled diabetes [25]. Further, similar to our findings, a meta-analysis of 13 cohort studies with 536,318 participants reported a significant inverse association between Mg intake and risk of type 2 diabetes [26]. Experimental studies suggest that magnesium supplementation may have benefits in people with diabetes. A meta-analysis of 21 RCTs showed that supplementation with Mg significantly improved insulin sensitivity and glucose tolerance [27]. Mg works as an insulin sensitizer by regulating the tyrosine kinase activity of insulin’s receptor [28]. In diabetes, insulin resistance decreases renal Mg reabsorption, resulting in urinary Mg wasting. Thus, people with diabetes may end up in a vicious circle in which hypomagnesemia enhances insulin resistance and insulin resistance causes hypomagnesemia [29]. Our findings, in addition to published data, suggest that magnesium supplementation may be needed in individuals with diabetes and may help in enhancing diabetes control.

Diabetes was associated with a 49% increase in the odds of having low iron, and the effect was worse in participants with uncontrolled diabetes. These findings are consistent with findings from some studies, where low blood iron marker levels were associated with diabetes [30]. However, many studies have studied multiple iron indices and described their associations with diabetes. Many of these studies have reported consistent associations between these iron indices and diabetes. In particular, increased ferritin and heme-iron intake were associated with diabetes in several meta-analyses [30], results that are in line with our study, but there are differences, as we only assessed serum iron. Future studies in this setting should assess the role of iron indices in both diabetes risk and diabetes control.

In the current study, low Zn levels were associated with diabetes, although with weak evidence against the null hypothesis, as shown by the *p*-value which suggests a lack of statistical significance. The weak evidence against the null hypothesis may be explained by the low sample size that was available for the zinc analysis. However, the effect size suggests that there is a possible clinically significant effect in the association between zinc and both the odds of diabetes and the odds of poor glycemic control. There are several in vitro [31,32,33] and in vivo studies [34] which suggest that Zn may have benefits in lowering the risk of diabetes and improving diabetes control. It is thought that Zn may exert these effects through its action on both β cell function and insulin action [23,24,25,26]. One systematic review and meta-analysis of 12 studies evaluated the effects of Zinc supplementation on glycemic control in patients with type 2 diabetes mellitus. In that meta-analysis, zinc supplementation was associated with a reduction in the fasting blood glucose (pooled weighted mean difference of 18.13 mg/dL, 95% CI: 33.85; 2.41; *p* < 0.05) and a reduction in the 2 h post-prandial blood sugar (pooled weighted mean difference of 34.87 mg/dL (95% CI: 75.44; 5.69)) in the participants who received zinc supplementation. Further, supplementation with zinc was also associated with a reduction in the mean HbA1c of 0.54% (95% CI: 0.86; 0.21) [35]. These findings suggest a protective effect of zinc in diabetes control and a possible preventive effect in the risk of diabetes. However, caution is required when interpreting these findings, as well as ours. Diabetes is a complex, multifactorial disease whose etiology is still not completely understood. For type 2 diabetes, the development of the disease may result from an interaction between genetic, epigenetic, environmental, and individual factors (such as excess adiposity), but the exact contribution of each of these factors to the overall diabetes risk is still not completely understood. Therefore, any interventions to reduce diabetes risk should be multifaceted, comprising different interventions targeting modifiable risk factors, and micronutrient and trace element supplementation may form a component of that multifaceted intervention. This is also true of interventions to enhance blood glucose control in those individuals already diagnosed with diabetes.

The current study has several limitations. Our study was a case–control study, but the measurements were carried out using a cross-sectional method; therefore, this did not enable us to establish temporality, i.e., that abnormalities in the nutrients were present before the development of diabetes or the start of the lack of diabetes control in a given individual. Another limitation is that we were not able to analyze data on physical exercise, diet, and alcohol consumption, which are important as they could be confounders. The level of vitamin D may also have affected both the level of micronutrients and diabetes risk and diabetes disease control. However, vitamin D deficiency is highly prevalent in Qatar (more than 70%, according to data from primary care centers [16]), and therefore, adjusting for this variable may not have influenced the results of the current study. Another limitation of this study is that the research data are from a population-based cohort where clinical data such as micro- and macrovascular complications of diabetes were not measured. These microvascular and macrovascular complications of diabetes include retinopathy, nephropathy, neuropathy, myocardial infarction, ischemic heart diseases, stroke, peripheral vascular diseases, and diabetic foot ulcers. The associations between micronutrients and trace elements may be altered in individuals with some of these diabetes complications. Therefore, we were not able to analyze the independent association between trace elements and diabetes in people with and without diabetes complications. Further, we could not distinguish between type 1 and type 2 diabetes mellitus since the QBB data did not make a clear distinction between the two. However, it is well known that at least 90% of diabetes in adults is type 2; therefore, this is most likely the case in our current study.

A further limitation of the present study is the significance of ethnicity in the association between micronutrients and trace elements and diabetes. There are many studies which have investigated how ethnicity and genetics may affect the pathogenesis and development of diabetes and insulin resistance [36]. However, in the current study, this limitation is not a particularly significant one as the Qatari population is relatively homogenous and so there was little to no ethnic variation between the Qataris in our study, which make up more than 85% of the QBB.

The current study is the first on this topic in Qatar, a country that has a relatively high prevalence of diabetes and where the prevalence of type 2 diabetes is expected to increase in the future. This study provides data about the association between micronutrients and diabetes in a relatively affluent population, where supplementation with these nutrients is relatively affordable. The current study also provides data on differences in the levels of these micronutrients between participants with controlled and uncontrolled diabetes, an area that has not been explored sufficiently in general, and in particular, in the MENA region. To determine whether these differences are a result of pathophysiological diabetes processes or due to nutritional supplementation, further and more robust research is required. Another area of further research would be experimental trials on whether Mg supplementation may result in better levels of diabetes control, as our observational data suggest.

## 5. Conclusions

In an affluent setting in the MENA region, diabetes was associated with low magnesium and low iron, and this association was worse in individuals with uncontrolled diabetes. Further research is required to understand whether supplementation with these nutrients could contribute to a reduction in diabetes risk, when included in multifaceted interventions on diabetes prevention. More research is also needed, especially from experimental studies, to investigate the effect of supplementation, especially with magnesium, in enhancing diabetes control in those individuals who already have diabetes.

## Figures and Tables

**Table 1:** Characteristics of participants by diabetes status.

Characteristic	No Diabetes	Controlled Diabetes	Uncontrolled Diabetes	*p*-Value
N	744	229	145	
Age in years, mean (SD)	40.3 (9.9)	43.8 (9.0)	45.3 (9.9)	<0.001
Sex, *n* (%)				
Female	404 (54.3%)	128 (55.9%)	71 (49.0%)	0.40
Male	340 (45.7%)	101 (44.1%)	74 (51.0%)	
Nationalty, *n* (%)				
Non-Qatari	107 (14.4%)	31 (13.5%)	24 (16.6%)	0.71
Qatari	637 (85.6%)	198 (86.5%)	121 (83.4%)	
Education, *n* (%)				
Primary school	465 (62.7%)	87 (38.0%)	98 (68.1%)	<0.001
Secondary/high school	127 (17.1%)	53 (23.1%)	14 (9.7%)	
Tertiary education	150 (20.2%)	89 (38.9%)	32 (22.2%)	
Employment status				
Employed	531 (71.4%)	168 (73.4%)	91 (62.8%)	0.069
Monthly salary (QAR)				
<10,000	134 (19.3%)	36 (16.7%)	37 (28.0%)	0.12
10–20,000	168 (24.2%)	51 (23.7%)	30 (22.7%)	
>20,000	393 (56.5%)	128 (59.5%)	65 (49.2%)	
HbA1c%, median (IQR)	5.2 (5.0, 5.4)	6.0 (5.5, 6.4)	7.8 (7.4, 8.9)	<0.001
BMI, kg/m^2^, mean (SD)	29.8 (5.6)	31.2 (5.2)	31.9 (5.7)	<0.001
Waist-to-hip ratio, mean (SD)	0.8 (0.1)	0.9 (0.1)	0.9 (0.1)	<0.001
Lipid profile				
Cholesterol Total, mmol/L, median (IQR)	5.0 (4.4, 5.6)	5.0 (4.3, 5.5)	5.0 (4.3, 5.7)	0.58
HDL-Cholesterol, mmol/L, median (IQR)	1.4 (1.1, 1.6)	1.2 (1.0, 1.5)	1.2 (1.0, 1.5)	<0.001
Triglyceride, mmol/L, median (IQR)	1.1 (0.8, 1.5)	1.4 (1.0, 1.9)	1.5 (1.0, 2.2)	<0.001
LDL-Cholesterol Calc, mmol/L, median (IQR)	3.0 (2.5, 3.5)	3.0 (2.4, 3.5)	2.9 (2.3, 3.5)	0.24
Dyslipidemia (yes), *n* (%)	249 (33.5%)	101 (44.1%)	67 (46.2%)	<0.001
Systolic BP, mmHg, mean (SD)	112.4 (13.3)	117.9 (14.3)	124.1 (17.5)	<0.001
Diastolic BP, mmHg, mean (SD)	67.4 (10.2)	70.5 (10.3)	74.0 (11.7)	<0.001
Hypertension (yes), *n* (%)	128 (29.0%)	28 (41.2%)	46 (54.1%)	<0.001
DM family history (yes), *n* (%)	195 (32.8%)	108 (65.9%)	75 (70.8%)	<0.001
Smoking statues, *n* (%)				
Non-smoker	27 (3.7%)	11 (5.0%)	4 (3.0%)	0.60
Current smoker	677 (92.0%)	198 (90.8%)	120 (90.2%)	
Ex-smoker	32 (4.3%)	9 (4.1%)	9 (6.8%)	
Multivitamin supplements, *n* (%)	239 (38.9%)	94 (51.1%)	45 (45.0%)	0.011

Percentages are column percentages. n is specified for variables with missing data. Abbreviations: BMI (body mass index), HbA1c (glycated hemoglobin), HDL (high-density lipoprotein), LDL (low-density lipoprotein), BP (blood pressure), SD (standard deviation), IQR (interquartile range). Dyslipidemia was defined as any participant with diagnosed dyslipidemia or any of the following: total cholesterol > 6.2 mmol/L, triglyceride > 2.3 mmol/L, low density lipoprotein (LDL) > 4.1, high-density lipoprotein (HDL) < 1 mmol/L and < 1.3 mmol/L for males and females, respectively. Hypertension was defined as any participants with diagnosed hypertension or either systolic blood pressure > 130 mmHg or diastolic blood pressure > 80 mmHg.

**Table 2:** Comparison of nutrient levels between participants with and without diabetes.

Factor	Level	No Diabetes	Diabetes	*p*-Value	Controlled Diabetes	Uncontrolled Diabetes	*p*-Value
N		744	374		229	145	
Iron, median (IQR)		14.6 (10.5, 18.7)	13.6 (9.7, 18.0)	0.036	13.4 (9.2, 18.0)	14.0 (10.0, 18.1)	0.110
Categorised iron, *n* (%)	Normal	603 (81.0%)	295 (78.9%)	0.390	178 (77.7%)	117 (80.7%)	0.540
	Low	141 (19.0%)	79 (21.1%)		51 (22.3%)	28 (19.3%)	
Magnesium (Mg), median (IQR)		0.8 (0.8, 0.9)	0.8 (0.8, 0.8)	<0.001	0.8 (0.8, 0.9)	0.8 (0.7, 0.8)	<0.001
Categorized magnesium, *n* (%)	Normal	578 (77.7%)	191 (51.1%)	<0.001	136 (59.4%)	55 (37.9%)	<0.001
	Low	166 (22.3%)	183 (48.9%)		93 (40.6%)	90 (62.1%)	
Zinc level, median (IQR)		12.4 (11.1, 13.7)	12.7 (11.1, 14.4)	0.290	12.3 (10.5, 14.7)	13.1 (11.6, 14.3)	0.270
Categorised zinc, *n* (%)	Normal	260 (85.8%)	46 (82.1%)	0.480	21 (75.0%)	25 (89.3%)	0.250
	Low	43 (14.2%)	10 (17.9%)		7 (25.0%)	3 (10.7%)	
Copper level, median (IQR)		17.6 (15.2, 20.5)	18.6 (16.3, 21.8)	0.099	18.6 (16.0, 20.9)	19.4 (16.4, 23.0)	0.200
Categorized copper level, *n* (%)	Normal	216 (71.3%)	45 (80.4%)	0.160	22 (78.6%)	23 (82.1%)	0.360
	Low	87 (28.7%)	11 (19.6%)		6 (21.4%)	5 (17.9%)	
Folate, median (IQR)		21.3 (16.0, 26.5)	23.5 (17.4, 28.7)	<0.001	23.5 (17.0, 28.5)	23.4 (18.2, 29.1)	0.295
Categorised folate, *n* (%)	Normal	744 (100.0%)	372 (100.0%)		228 (100.0%)	144 (100.0%)	
Vitamin B12, median (IQR)		243.0 (194.0, 326.0)	239.0 (186.0, 324.5)	0.710	224.0 (176.0, 299.0)	263.0 (206.0, 371.5)	0.014
Categorised vitamin B12, *n* (%)	Normal	678 (91.3%)	338 (90.9%)	0.830	203 (89.0%)	135 (93.8%)	0.290
	Low	65 (8.7%)	34 (9.1%)		25 (11.0%)	9 (6.3%)	

Abbreviations: Fe (iron), Mg (magnesium), Zn (zinc), Cu (copper), SD (standard deviation), IQR (interquartile range). The normal ranges for the micronutrients are as follows [20]: Mg (0.66 to 1.07 mmol/L), Fe (9 to 27 μmol/L), Zn (11.5 to 21.4 μmol/L), Cu (15.7 to 31.4 μmol/L), folate (4.1 to 20.4 nmol/L), vitamin B12 (147.5 to 590 pmol/L).

**Table 3:** Association between controlled diabetes and micronutrients—multivariable multinomial logistic regression.

	All Diabetes			Controlled Diabetes			Uncontrolled Diabetes		
Micronutrient	OR	*p*-Value	95%CI	OR	*p*-Value	95%CI	OR	*p*-Value	95%CI
Mg	3.32	0.000	2.47–4.47	2.47	0.000	1.75–3.48	5.57	0.000	3.65–8.52
Fe	1.49	0.034	1.03–2.16	1.38	0.141	0.90–2.11	1.75	0.047	1.01–3.03
Zn	1.49	0.336	0.66–3.34	2.47	0.063	0.95–6.41	0.62	0.531	0.14–2.77
Cu	0.68	0.382	0.28–1.62	1.17	0.784	0.38–3.59	0.35	0.125	0.09–1.34
Vitamin B12	1.17	0.508	0.74–1.84	1.37	0.231	0.82–2.27	0.84	0.641	0.40–1.76

Abbreviations: Fe (iron), Mg (magnesium), Zn (zinc), Cu (copper), OR (odds ratio), CI (confidence interval). Adjusted for age, gender, BMI, smoking, and income.

## Data Availability

The data used in this study are the property of the Qatar Biobank and are available at https://researchportal.qatarbiobank.org.qa/ (14 September 2023).

## Abbreviations

Fe (iron), Mg (magnesium), Zn (zinc), Cu (copper), MENA (Middle East and North Africa), HbA1c (glycated hemoglobin), BMI (body mass index), HDL (high-density lipoprotein), LDL (low-density lipoprotein), BP (blood pressure), OR (odds ratio), CI (confidence interval), β (beta coefficient), RCT (randomized control trial).
